# The Triform algorithm: improved sensitivity and specificity in ChIP-Seq peak finding

**DOI:** 10.1186/1471-2105-13-176

**Published:** 2012-07-24

**Authors:** Karl Kornacker, Morten Beck Rye, Tony Håndstad, Finn Drabløs

**Affiliations:** 1 Division of Sensory Biophysics, Ohio State University, Columbus, OH, USA; 2 Department of Cancer Research and Molecular Medicine, Norwegian University of Science and Technology (NTNU), P.O. Box 8905, NO-7491, Trondheim, Norway

**Keywords:** ChIP-Seq, Peak finding, Benchmark, Repeats

## Abstract

**Background:**

Chromatin immunoprecipitation combined with high-throughput sequencing (ChIP-Seq) is the most frequently used method to identify the binding sites of transcription factors. Active binding sites can be seen as peaks in enrichment profiles when the sequencing reads are mapped to a reference genome. However, the profiles are normally noisy, making it challenging to identify all significantly enriched regions in a reliable way and with an acceptable false discovery rate.

**Results:**

We present the Triform algorithm, an improved approach to automatic peak finding in ChIP-Seq enrichment profiles for transcription factors. The method uses model-free statistics to identify peak-like distributions of sequencing reads, taking advantage of improved peak definition in combination with known characteristics of ChIP-Seq data.

**Conclusions:**

Triform outperforms several existing methods in the identification of representative peak profiles in curated benchmark data sets. We also show that Triform in many cases is able to identify peaks that are more consistent with biological function, compared with other methods. Finally, we show that Triform can be used to generate novel information on transcription factor binding in repeat regions, which represents a particular challenge in many ChIP-Seq experiments. The Triform algorithm has been implemented in R, and is available via http://tare.medisin.ntnu.no/triform.

## Background

Chromatin immunoprecipitation combined with high-throughput sequencing (ChIP-Seq) is currently the method of choice for genome-wide mapping of binding sites for transcription factors (TFs) on DNA [1-3]. This is achieved by using DNA fragmentation after DNA-binding proteins have been cross-linked to the genome. The fragmentation is followed by selection of fragments bound by the TF of interest, using an antibody targeting this factor. The selected genomic fragments are then released, sequenced, and mapped to a reference genome. There, the genomic locations bound by the TF will be enriched with matching sequencing reads.

An essential step in the analysis of ChIP-Seq data is the genome-wide identification of enriched regions. Several software tools have been developed to perform this task [4,5], but benchmarking has demonstrated that there is room for improvement in the existing approaches [6]. There are alternative definitions of what constitutes a peak, and useful characteristics of ChIP-Seq profiles may not be fully utilized. A typical example is the shift property [7], which occurs because the full sequence fragments, typically with an average length around 200 bp, are sequenced for only 25–50 bp from each side. Other examples are the use of independent control samples [8], separation of overlapping enrichment profiles [9,10], or optimal use of statistical approaches to separate signal from noise [11]. Our conclusion in a previous study [6] was that different programs focus on different characteristics of the ChIP-Seq data, but that no program takes all the characteristics into account. Though several programs achieve a high coverage of true enrichment profiles, the trade-off has often been an intolerably high false discovery rate (FDR).

A major limitation in the development of improved approaches has been the lack of proper benchmarks to compare the performance of different methods [12]. Because of this, different strategies for performance evaluation have been used, the most common being motif occurrences in the proximity of the ChIP-Seq peaks [4,5,8,13], and overlap with experimentally confirmed qPCR sites [4,5]. However, both evaluation methods have important limitations [6,12], and when looking at a limited number of binding regions, the preferred evaluation method remains visual assessment of local enrichment profiles in a genome browser. To compensate for the lack of benchmarks in ChIP-Seq analysis, we have previously manually curated a benchmark data set based on visual inspection of ChIP-Seq profiles from three different TFs: NRSF (also known as REST), SRF, and MAX [6]. Individual regions were visually assessed relative to relevant criteria, including peak-like shape, peak shift, lack of signal in control sample, and motif occurrence in peak region, and the regions were classified either as real peaks or as noise or artifacts. The idea was that such a benchmark can be used to evaluate and improve both new and existing efforts for the automatic analysis of ChIP-Seq data for TFs.

In this study, we present an improved approach for automatic identification of peaks in ChIP-Seq enrichment profiles, called the Triform method. Triform uses robust genome-wide statistical tests to detect three different forms of peak-like enrichment profiles, taking advantage of the profile characteristics mentioned above. Overfitting is precluded by the fact that Triform is based on model-free statistical tests and uses a minimal number of preset parameters based on the general properties of the ChIP-Seq data sets. The Triform algorithm is model free in the sense that it relies on the Hoel test [14] which is fully nonparametric, i.e. free from any assumed relationships or fitted parameters. In particular, the Hoel test is free from any assumed background model and is therefore more robust than model-based tests, which depend on locally uniform background models and fitted background parameters. When evaluated on the benchmark data set of peak profiles, Triform outperformed both newly developed and previously evaluated programs for the automatic detection of enrichment profiles, for all three TFs studied. The good performance of Triform was further confirmed using tests on motif enrichment in significant peak regions.

Since TFs often co-regulate genes that are involved in specific biological processes, we would expect such processes to be overrepresented in the annotation of genes associated with regions for TF binding from the ChIP-Seq experiment. We therefore used statistical overrepresentation analysis on peak sets from the main peak-finding programs that are compared here, and showed that peak sets from Triform in most cases give the most significant overrepresentation of relevant annotation terms.

To illustrate the potential of improved peak finding to generate novel information, we further analyzed the DNA sequence motifs identified *de novo* within the Triform SRF peak regions. In addition to the canonical SRF motif, we discovered a significant co-occurrence of SRF, ELK1, and NFY motifs within LTR/ERV1/MER57 repeats, and these particular repeats were significantly co-located with genes associated with cancer. This exemplifies how optimal identification of peak regions may generate novel information.

## Results and discussion

### The Triform algorithm

The main problem with peak finding in ChIP-Seq data for TFs is the reliable differentiation between peaks and noise. Many algorithms define a peak as a region of significantly elevated coverage of sequencing reads. Consequently, such algorithms tend to accept false positives in the form of noisy plateaus, *i.e.*, wide regions of elevated coverage lacking a distinctive core sub-region and lacking a well-defined shift between coverage profiles on opposite strands. Such regions are expected to be present, to some extent, in any ChIP-Seq data set.

The Triform algorithm defines a peak as a region with a significantly negative mean second derivative of the coverage profile, using model-free test statistics developed by Hoel [14]. Such regions have inherently limited width, peak sub-regions are directly identified, and these sub-regions can be used to test for well-defined shifts between overlapping profiles on opposite strands. The test can also handle overlapping peaks. Consequently, the Triform algorithm is able to reject false positive noisy plateaus, thereby increasing specificity with little or no loss of sensitivity. This is used in combination with other important features and data, in particular control data and biological replicates. Additional sequencing of control data emulating tag enrichment profiles created without targeting any specific TF has become standard in ChIP-Seq, and may be used to improve the separation of true peaks from noise and artificial enrichment. The same is true for biological replicates.

#### Calculation of model-free test statistics for local peak-like forms on each strand

At each strand location *x* the raw ChIP-Seq coverage $C\left(x\right)$ is formally regarded as a Poisson variate with parameter $\lambda \left(x\right)=E\left[C\left(x\right)\right]$. The formal Poisson model leaves the Poisson parameter unspecified and does not assume homogeneity across technical replicates, but does imply that any summation of independently measured Poisson variates yields another Poisson variate.

For read width *w* and fixed parameter δ>w (see Table 1), the three coverage values $\left\{C\left(x-\delta \right),C\left(x\right),C\left(x+\delta \right)\right\}$ at any location *x* are formally regarded as independently measured Poisson variates with parameters $\left\{\lambda \left(x-\delta \right),\lambda \left(x\right),\lambda \left(x+\delta \right)\right\}$.

**Table 1:** Triform parameters

**Parameter**	**Description**
*w*	read width, symmetrically extended to a fixed value (default 100 bp)
*δ*	fixed spacing between central and flanking locations (must be > *w*, default 150 bp)
*r*	actual ratio between control and ChIP-Seq library sizes
min.z	fixed minimum upper-tail z-value (default corresponds to standard normal *p* = 0.1)
min.n	fixed minimum number of bp (peak width) in peak-like region (default 10 bp)
min.er	fixed minimum local enrichment ratio (default 3/8 quantile of the enrichment ratio)
min.lag	fixed minimum inter-strand lag between peak coverage distributions (default 10 bp)

**Figure 1:**
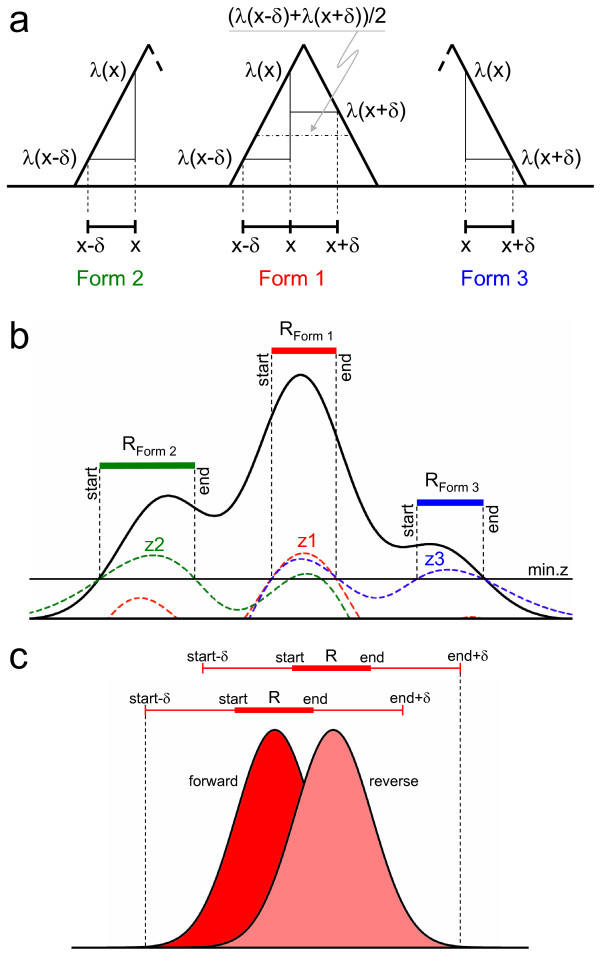
**Triform approach to peak definition.** Key aspects of the Triform algorithm. Part **a**) shows the definition of the three alternative peak-shape hypotheses Forms 1, 2, and 3 using very simplified peaks. Part **b**) shows this on a more realistic peak shape, using the min.z cut-off on the *z*1, *z*2, and *z*3 test statistics to define the start and end of the regions R of each peak-like form. Part **c**) shows how strand lag for a peak pair on the forward (red) and reverse (pink) strand is estimated, using the *δ* parameter to define an extended region. Triform uses cross-correlation on this region to estimate the optimal lag, and compares this with the minimum inter-strand lag min.lag.

The following three alternative hypotheses are tested for local peak-like forms (see Figure 1a):

(1) $$2\lambda \left(x\right)>\lambda \left(x-\delta \right)+\lambda \left(x+\delta \right)$$

(2) $$\lambda \left(x\right)>\lambda \left(x-\delta \right)$$

(3) $$\lambda \left(x\right)>\lambda \left(x+\delta \right)$$

The above three hypothesized peak-like forms will be annotated as Form 1, Form 2, and Form 3 peaks, respectively. In Form 1 peaks, the peak coverage is higher than the average flanking coverage, while in Form 2 and Form 3 peaks the peak coverage is higher than the left- or right-flanking coverage, respectively (Figure 1a).

The following three model-free test statistics for peak-like forms are based on equation (11) of Hoel [14]. These statistics have approximately standard normal distributions even for arbitrarily low positive total counts.

(4) $$z1\left(x;\delta \right)=\frac{2C\left(x\right)-\left(C\left(x-\delta \right)+C\left(x+\delta \right)\right)}{\sqrt{2\left(C\left(x-\delta \right)+C\left(x\right)+C\left(x+\delta \right)\right)}}$$

(5) $$z2\left(x;\delta \right)=\frac{C\left(x\right)-C\left(x-\delta \right)}{\sqrt{C\left(x-\delta \right)+C\left(x\right)}}$$

(6) $$z3\left(x;\delta \right)=\frac{C\left(x\right)-C\left(x+\delta \right)}{\sqrt{C\left(x+\delta \right)+C\left(x\right)}}$$

#### Calculation of model-free test statistics for local enrichment on each strand

At each strand location *x* the raw coverage $B\left(x\right)$ of the control sample is formally regarded as a Poisson variate with Poisson parameter $\mu \left(x\right)=E\left[B\left(x\right)\right]$. For a given ratio *r* between the control and ChIP-Seq library sizes (see Table 1), the tested hypothesis of local enrichment is:

(7) $$r\cdot \lambda \left(x\right)>\mu \left(x\right)$$

The model-free test statistic for local enrichment, $z4\left(x\right)$, is calculated according to (8). The local enrichment ratio, $ER\left(x\right)$, is calculated according to (9).

(8) $$z4\left(x\right)=\frac{r\cdot C\left(x\right)-B\left(x\right)}{\sqrt{r\left(B\left(x\right)+C\left(x\right)\right)}}$$

(9) $$ER\left(x\right)=\frac{1+r\cdot C\left(x\right)}{1+B\left(x\right)}$$

#### Calculation of local inter-strand lags between overlapping peaks on opposite strands

Figure 1c illustrates the range of locations involved in the calculation of the local inter-strand lag between two overlapping Form 1 peak regions on opposite strands. The range starts at the $\left(start-\delta \right)$ position on the forward strand and ends at the $\left(end+\delta \right)$ position on the reverse strand. The optimal lag is taken as the one that maximizes the cross-correlation between the forward and reverse coverage distributions within the specified range.

#### Procedure for detecting local peak-like forms

Parameters and default parameter settings are shown in Table 1. The parameters are fixed values to avoid overfitting. The default values have been chosen to reflect well-known properties of ChIP-Seq data. For example, the default value of the spacing parameter *δ* is 150 bp, which is comparable to the length of DNA around a nucleosome. The cut-off for the minimum local enrichment ratio min.er is needed because non-specific ChIP-Seq coverage is often significantly higher than the coverage in the control sample. Currently the min.er cut-off value is calculated for each strand as the 3/8 quantile of the enrichment ratios in significantly enriched Form 1 peaks. The choice of quantile cut-off value does not seem to be critical because the 1/4 and 1/2 quantiles were nearly equal in all tested data sets.

The necessary and sufficient conditions to detect a local peak-like form within a region R on one strand are:

The number of base pairs in region R (peak width) exceeds a given minimum min.n.

For every location *x* in region R: $zf\left(x;\delta \right)>$min.z, where *zf* denotes one of the standard normal test functions *z*1, *z*2, and *z*3 defined by equations (4)–(6) above (see Figure 1b), and min.z is a fixed minimum upper-tail z-value cut-off for Hoel tests.

For every location *x* in region R: $z4\left(x\right)>$min.z.

For location $x_{0}$ at the center of region R: $ER\left(x_{0}\right)>$min.er.

If biological replicates are available, then for every replicate *k* and every location *x* in region R: $zf\left(x;k,\delta \right)>0$ and $z4\left(x;k\right)>0$.

The criteria to detect local peak-like forms on both strands are:

For each of the three peak forms accept only those detected peak regions that overlap exactly one detected peak region on the opposite strand.

Accept only those pairs of overlapping peak regions whose local inter-strand lag exceeds a given minimum min.lag (see Figure 1c).

Reject any redundant Form 2 or Form 3 peaks that overlap a Form 1 peak.

Merge any overlapping Form 2 and Form 3 peaks into Form 1 peaks.

#### Implementation of the Triform algorithm

The Triform method has been implemented in R using the IRanges package. For each detected peak region, the peak position (PEAK.LOC) is reported as the midpoint of the range, and the peak significance (PEAK.NLP) is reported as the sum of the Negative Log10 (P) (NLP) values for the test statistics calculated according to equations (4)–(6). This is proportional to the Fisher’s *χ*^2^ statistics for the combined null hypothesis that all individual null hypotheses are true. Details of the implementation can be found in Additional file 1. The Triform algorithm has been implemented in R, and is available via http://tare.medisin.ntnu.no/triform. It has been submitted to Bioconductor for inclusion in the Bioconductor package [15].

### Triform outperforms other methods on a manually curated ChIP-Seq benchmark

**Figure 2:**
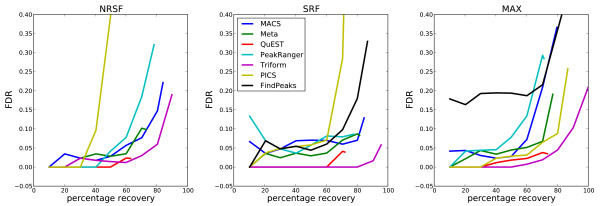
**Peak profile benchmark results.** Triform outperforms other methods on ChIP-Seq peak profile benchmarks for the TFs NRSF, SRF, and MAX. Coverage, which is the percentage of true peaks recovered in the evaluated regions, is plotted against the false discovery rate (FDR) for each method evaluated. The peak lists from each method are first sorted according to the peak scores from that program. The corresponding coverage and FDR are then calculated as the list is traversed starting at the highest scoring peak. In general, Triform has the lowest FDR for medium-to-high coverage. To emphasize the details at the lower FDR levels, the results from TPic are not shown because of the high FDRs produced by this method. The results from FindPeaks for NRSF are not shown due to the low total number of regions that were recovered (389), giving a total coverage of only 7%.

We evaluated the performance of Triform on the manually curated ChIP-Seq benchmark for peak-like enrichment profiles, created by Rye et al. [6] (see Figure 2). The performance of Triform was compared with that of seven other programs for ChIP-Seq peak identification: QuEST, MACS, the Meta approach by Rye et al., PICS, FindPeaks, PeakRanger, and TPic. QuEST and MACS are popular, and have both performed well in previous program evaluations [4,5]. Meta was the method used by Rye et al. during the original benchmark, and is a combination of outputs from the programs MACS and SISSRs [7]. PICS uses a Bayesian approach to identify binding events [16]. FindPeaks tests for local peak-like coverage distributions [9] and is the tool that is currently most similar to the Triform algorithm. PeakRanger and TPic are recent additions to the family of ChIP-Seq peak-identification programs, and have, to our knowledge, not yet been independently evaluated.

Triform clearly outperformed all these methods on peak finding, for all three TFs that were analyzed, both with respect to coverage, which is the percentage of true peaks identified, and the FDR. Most importantly, Triform recovered 80% of the peaks at a 0.05 FDR level. For NRSF and SRF, Triform kept the FDR at an acceptable 0.1 when the coverage went beyond 90%. For the more challenging MAX data set, the FDR was somewhat higher when the coverage exceeded 90%. However, given the differences in binding patterns for the three factors, especially exemplified by MAX that includes many partly overlapping peaks, Triform showed consistently good performance for all factors.

### Triform shows good performance on a motif enrichment benchmark

**Figure 3:**
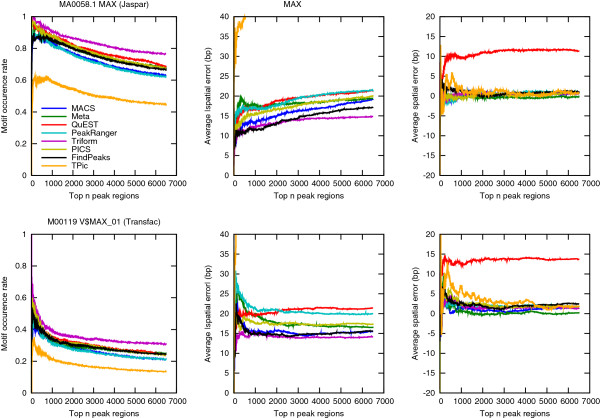
**Motif enrichment benchmark results.** Triform shows good performance on the motif enrichment benchmark. The figure shows motif occurrence rate of the Jaspar MAX motif (MA0058.1) in peak regions (±150 bp around predicted peak summit) sorted according to significance. Also shown is the average absolute and relative spatial error of the motif position relative to the predicted peak summit. If more than one significant motif was found in a given region, only the motif closest to the predicted peak summit was used for statistics. Please see Additional file 2 for full results.

A frequently used benchmark approach is to test genomic regions from a peak finder for binding site motifs for the TF sampled in the ChIP-Seq experiment, assuming that the best performing peak finders will identify peak regions with a high occurrence rate of relevant motifs. Here we used the approach described for example in the evaluation of PICS [16]. Output from each peak finder was sorted according to significance, and for the top *n* peak regions a representative binding site motif for the given TF was used to scan each region for significant binding sites. Please see Methods for details. Both the fraction of regions containing binding sites, and the average distance from the estimated peak summit was estimated. Results for MAX using the MAX binding site motif from Jaspar [17] is shown in Figure 3; full results for all three TFs are found in Additional file 2: Figure S1 to S3.

The good performance of Triform on the motif enrichment benchmark confirms that the superior performance on the peak finding benchmark is consistent with a high biological relevance. Triform also seems to have the best overall performance with respect to finding the optimal peak summit. Apart from this, the difference between methods with respect to motif enrichment is relatively small, except for a couple of methods with sub-optimal performance, and the relative performance of all methods seems to vary with data set and motif model. This test is easily biased for example by the choice of motif description, the relative frequency of indirect binding of TFs, and possible lack of specificity of the antibody used in the ChIP-Seq experiment. This is therefore a more indirect test of peak finder performance than the peak profile benchmark described in the previous sub-section.

### Triform peaks generally show the most significant statistical association with relevant biological processes

As an evaluation of the general quality of the peak set defined by selected methods, the highest-scoring peaks were submitted to GREAT [18] for statistical overrepresentation analysis. As the total number of peaks varied, depending on both the data set and prediction method, a fixed number of peaks representing only those with highest score was used in each case. This was to make the conditions as similar as possible for the different tools. These peak lists were submitted to GREAT, which associates peaks (entered as genomic regions) with genes, and test gene annotations for statistically overrepresented terms relative to a general genomic background. Selected properties of the overrepresentation analysis were evaluated and are summarized in Table 2. These features were ranked high by all or most of the methods, and represent in general known processes or features associated with each of the TFs.

**Table 2:** **Results of statistical overrepresentation analysis with GREAT**^**a**^

**Method**	**MACS**	**Meta**	**QuEST**	**PeakRanger**	**TPic**	**Triform**
**NRSF**						
Peaks total (2894 common)	9802	4750	3574	6123	37514	10583
Peaks used (1993 common)	3500	3500	3500	3500	3500	3500
Genes	4017	4017	4105	3956	3866	4033
GO – Neurotransmitter transport	1.6 × 10^-10^	6.0 × 10^-11^	1.2 × 10^-10^	2.5 × 10^-6^	5.2 × 10^-7^	**9.3 × 10**^**-13**^
TF – REST motif	1.1 × 10^-26^	4.6 × 10^-25^	5.4 × 10^-19^	1.4 × 10^-25^	5.6 × 10^-23^	**1.1 × 10**^**-31**^
**SRF**						
Peaks total (608 common)	4012	1553	1383	1320	111305	9555
Peaks used (310 common)	1300	1300	1300	1300	1300	1300
Genes	1930	1887	1855	1816	1565	1837
GO – Actin cytoskeleton	1.8 × 10^-7^	**2.6 × 10**^**-9**^	1.2 × 10^-6^	2.1 × 10^-8^	-	3.8 × 10^-9^
TF – SRF-motif	6.0 × 10^-9^	3.6 × 10^-12^	2.4 × 10^-11^	3.6 × 10^-12^	2.4 × 10^-11^	**2.3 × 10**^**-18**^
**MAX**						
Peaks total (3306 common)	21866	10323	6556	9383	61021	15137
Peaks used (2014 common)	6500	6500	6500	6500	6500	6500
Genes	6139	6393	6626	6283	6276	6668
GO – ncRNA processing	4.3 × 10^-18^	5.9 × 10^-14^	2.0 × 10^-18^	**3.9 × 10**^**-22**^	3.1 × 10^-16^	1.5 × 10^-12^
GO – DNA duplex unwinding	**9.7 × 10**^**-43**^	3.9 × 10^-14^	-	1.6 × 10^-5^	1.1 × 10^-25^	-
TF – MYC motif	2.5 × 10^-12^	2.7 × 10^-14^	5.3 × 10^-16^	-	-	**1.7 × 10**^**-17**^
TF – E2F1 motif	2.7 × 10^-12^	1.8 × 10^-12^	-	**7.3 × 10**^**-15**^	5.4 × 10^-13^	2.9 × 10^-11^
TF – c-MYC from ChIP-chip	2.1 × 10^-36^	2.8 × 10^-35^	8.7 × 10^-48^	3.9 × 10^-36^	1.8 × 10^-46^	**7.8 × 10**^**-58**^

^a^The table shows (for each TF) the total number of peaks identified by each peak-finder (including the number of peaks common to all methods), the number of peaks used for GREAT analysis (including the number of common peaks), the number of associated genes identified by GREAT, and the binominal FDR Q-values identified by GREAT for the selected GO and TF-associated terms. The smallest Q-value for each term is shown in bold.

The analysis of NRSF/REST was based on the top 3500 peaks from each method, and the peak regions showed an overlap from 94% to 64% among the different methods. GREAT associated on average 4000 genes with these peak regions. Overrepresentation analysis focused on the gene ontology (GO) feature “Neurotransmitter transport” and TF feature “REST motif” from Predicted Promoter Motifs. The highlighted GO feature is supported by previous publications [19,20]. For both features, Triform showed the most significant overrepresentation.

The analysis of SRF was based on 1300 regions, with an inter-method overlap between 77% and 39%. GREAT associated on average 1800 genes with these peak regions. Overrepresentation analysis focused on the GO feature “Actin cytoskeleton”, which is supported, for example, by the findings of Sun et al. [21], and on the TF feature “SRF motif”. Here, Meta showed the most significant overrepresentation of GO features, whereas Triform was best on the TF feature. However, the difference between Meta and Triform was small and barely significant.

Finally, the analysis of MAX was based on 6500 regions, with an inter-method overlap between 75% and 49%. On average, 6300 genes were associated with these peak regions. Here, the variation among the methods with respect to overrepresented features was much larger. Therefore, several features were compared: two GO features (“ncRNA processing” and “DNA duplex unwinding”) and three TF features (“MYC motif” and “E2F motif” from Predicted Promoter Analysis, and “c-MYC from ChIP-chip” from Perturbation). The interaction between MAX and MYC is well documented (e.g., [22]). The interaction between MYC (and therefore probably MAX) and E2F has also been demonstrated previously [23]. The relevance of the GO properties is supported for example by [24], which discusses the role of MYC in the regulation of ncRNA expression, and by [25] on the role of MYC in replication. In this analysis, Triform showed the best performance on two of the TF features (see Table 2 for details).

Because of the somewhat inconsistent results for MAX, two additional tests were performed on this data set. First, a smaller data set was tested, using only the 3500 most significant peaks. Second, a moving window approach was used to select the peak sets, shifting the window 1000 peaks down the ranked list for each test. The rationale behind this was to test whether this data set contained a mixed signal, where the weaker peaks represented regulation of other processes (for example through a co-factor), compared with the stronger peaks. However, neither of these tests provided clarification, and in general, the significance was reduced.

In summary, overrepresentation analysis shows that the Triform method tends to identify peaks that are significantly associated with relevant biological processes.

### Triform peak regions facilitate the detection of co-occurring SRF/ELK1/NFY motifs in LTR/ERV1/MER57 repeats

It has recently been shown [26] that a significant fraction of STAT1-binding sites are found in the primate-specific MER41 repeat. This illustrates the potential role of repeat regions in gene regulation [27], and makes it relevant to investigate other TFs similarly. However, analysis of repeat regions in ChIP-Seq data may be challenging. The mapping of reads from repeat regions is often not unique, potentially leading to more noisy peaks in these regions. This makes it beneficial to use a peak-finder with high sensitivity and specificity for non-optimal peak shapes.

We used the SRF ChIP-Seq set, as this TF is known to associate with viral long terminal repeats (LTRs) [28]. We focused the analysis on 1510 Form 1 Triform peaks with PEAK.NLP > 12. We initially used 2410 low-significance TPic SRF peaks as negative control regions, assuming that, given the high number of peaks returned by TPic, these regions were most likely to be SRF-binding-like regions without significant regulatory importance (at least in this context). However, using the 2522 least significant Triform SRF peaks (PEAK.NLP < 4) gave a similar result, and was used for the analysis shown here. We then used CisFinder for *de novo* motif discovery in the Triform peak regions (±150 bp around the peak summit), and compared these motifs with known motifs.

**Figure 4:**
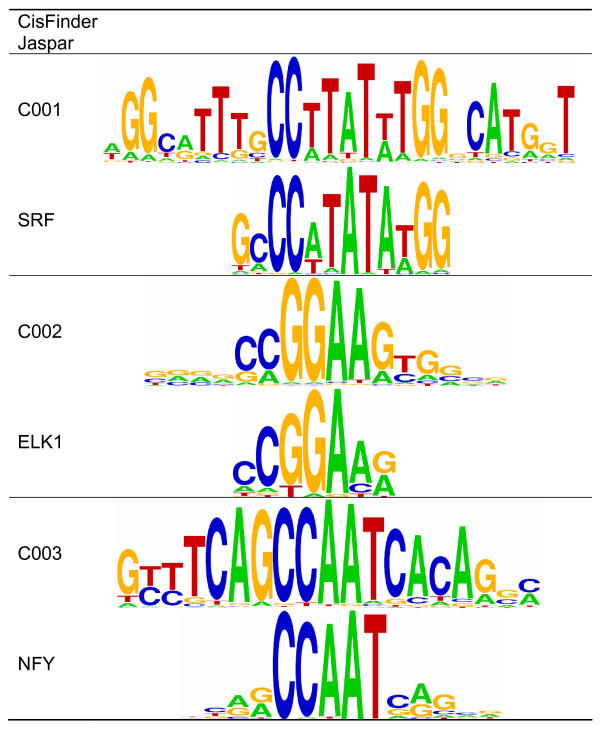
**CisFinder motifs from Triform regions of the SRF ChIP-Seq data set.** The figure shows the output from CisFinder, together with the corresponding Jaspar motifs for SRF (MA0083), ELK1 (MA0028), and NFY (MA0060). The sequence logos were generated with WebLogo.

CisFinder found three significantly overrepresented motifs (Figure 4): an SRF-like motif known as the CArG box, an ELK1-like motif (CCGGAA), and an NFY-like motif (CCAAT). The most likely identity of these motifs was determined by comparison of the position-specific frequency matrices against the Jaspar database using the T-Reg Comparator, identifying the most similar Jaspar motifs to be MA0083, MA0028, and MA0060, respectively. The co-occurrence of CArG box and ELK1-like motifs is well documented [29,30]. In addition, the co-occurrence of the SRF and NFY motifs has been observed previously [31-33]. The SRF/ELK1/NFY co-occurrence was strongly associated with LTRs of the medium reiteration sequence type (MER) from endogenous retrovirus (ERV) transposons (LTR/ERV1/MER57). Of the 9555 SRF regions from Triform, 124 regions overlapped with MER57 repeats (with an average overlap of 91%), and 117 of these contained at least two significant binding sites for TFs (from the CisFinder analysis), which is highly significant (*p* = 1.2 × 10^-65^ according to a Fisher exact test). Submitting these MER57 regions to GREAT with a general genome-wide list of MER57 regions as background showed that the subset of repeat regions identified here co-locates with genes significantly associated with cancer (thyroid and gastric cancer, with FDR Q-values of 1.9 × 10^-2^ and 3.6 × 10^-2^, respectively). GREAT also indicated that this subset of MER57 regions is located in more gene-rich regions than the general background set, as the foreground set of MER57 regions on average was associated with 1.7 genes, compared with 0.5 genes for the full set of MER57 regions.

There is a potential risk of bias in *de novo* motif discovery involving multiple repeat regions, as any motif associated with repeats will be highly significant. Although the fraction of MER57 repeats is low (1.3% of the total set), the CisFinder motif discovery was repeated with the same negative background regions, but using a positive set where all regions overlapping MER57 repeats had been removed. The final SRF-like and ELK1-like motifs were almost identical to the ones identified on the full sequence set. The NFY-like motif was also very similar, but was found at a lower frequency. This shows that the presence of MER57 repeats had only a minor effect on CisFinder motif discovery, in particular for the SRF and ELK1-like motifs.

It has long been suspected that LTRs may play a role in carcinogenesis [34]. The most likely mechanism seems to be that LTRs act as extra promoters leading to modified (possibly aberrant) expression of potential oncogenes [35,36]. In that respect, it is interesting that the MER57 repeats identified here co-locate with tumor-associated genes, where they may act as additional promoters. It has also recently been shown that tumor-associated microvesicles contain high levels of retrotransposon RNA transcripts [37], indicating that retroelement expression itself may play a role in carcinogenesis [38]. It is too early to say whether the subset of MER57 repeats identified here is involved in any of these processes. However, our analysis shows that using an approach for peak finding with improved sensitivity and specificity can generate interesting hypotheses for further testing.

## Conclusions

It is challenging to evaluate ChIP-Seq peak-finding methods because we normally do not know the true solution to a given experiment. The intensity of a given peak reflects the frequency of interaction between the TF and the genomic region, and thus the interaction strength. It is therefore tempting to focus on the strong peaks, assuming that these represent the most important regulatory interactions. However, this may be misleading for several reasons. First, the apparent binding strength may be affected in many different ways, including post-translational modifications and protein-protein interactions involving the relevant TF. The formation of cis-regulatory modules, including indirect binding, may also affect the efficiency of immunoprecipitation and pull-down of the relevant genomic fragments. The mapping of reads to the reference genome may also be biased, for example by differences between the sequenced genome and the reference genome used for read mapping. This means that we also have to include minor peaks in the analysis, which makes the peak finding more challenging. In addition, many protein-protein interactions lead to more complicated peak shapes, making the problem even more challenging.

Triform attempts to improve peak finding by identifying and using essential features of typical ChIP-Seq peaks, including peak shape and sequencing-induced peak shifts. This has been implemented in a rigorous model-free statistical framework, making the classification both robust and sensitive. In particular, Triform achieves greater sensitivity, specificity, and control of the FDR than other methods by utilizing the Hoel test to detect significant Poisson inhomogeneities, as could be seen in its comparison with, for example, FindPeaks.

As Triform gains performance by addressing specific properties of ChIP-Seq peaks, it could be argued that Triform may lead to model overfitting by favoring features that are important mainly in the benchmark set. However, the selected features represent completely general ChIP-Seq peak features, and the number of parameters in the Triform implementation has been reduced to a minimum. In combination with the statistical framework, we believe that this makes the algorithm more resistant to overfitting.

The excellent performance of Triform has been confirmed by the tests described here, including benchmarking, statistical overrepresentation analysis, and motif discovery for novel motifs. In all cases, the evaluation was limited by the fact that no perfect solution is available as a reference. However, in our opinion, all these tests indicate that Triform is at least as good as any of the methods it has been compared with, and in many cases is better.

However, it is important to be aware that although the Triform approach uses a quite general framework, the implementation is adapted to peak finding in ChIP-Seq experiments for TFs. It is likely that application to other types of ChIP-Seq experiments, for example for chromatin structure, will require a modified approach.

## Methods

### Data sets

The ChIP-Seq benchmark data set is based on sets of manually evaluated regions for three TFs: NRSF/REST, SRF, and MAX [6]. All the original ChIP-Seq tag files and results from the manual evaluations were downloaded from http://tare.medisin.ntnu.no/chipseqbenchmark/.

The full data sets for NRSF/REST, SRF, and MAX were downloaded from the ENCODE [39] repository of the UCSC Genome Browser [40] as specified in [6], and these data were used for peak finding for motif enrichment benchmark and statistical overrepresentation analysis. Identification of co-occurring peaks in repeats was based on the SRF data set. The list of MER57-type repeats for hg18 was downloaded from the UCSC Genome Browser.

### Software tools

The following program versions were used in the evaluations: QuEST v2.4 [41], MACS v1.4.0 [8], PICS v1.0.6 [16], FindPeaks v4.0 (as part of the Vancouver Short Read Analysis Package v4.0.16) [9], PeakRanger v1.02 [42], TPic from January, 2011 [10], and the Meta approach from March, 2011 [6]. All programs were run with default parameters, and the peaks from each program were sorted according to the score given in that program. Exceptions are TPic, and to some extent FindPeaks. TPic does not return any score for its final peaks. We therefore sorted the peaks from TPic according to tag enrichment. FindPeaks returns identical score values for large sub-sets of peaks, and these sub-sets were therefore subsequently sorted according to tag enrichment, in order to rank all peaks. For all programs, including Triform, samples and replicates were pooled into a single sample before analysis. To make the peak-lengths returned by the different programs comparable, we used the peak summit with a 150-bp extension in both directions as peak regions for all programs. A region length of 300 bp is in accordance with the resolution offered by most ChIP-Seq data.

Statistical overrepresentation analysis of predicted peak sets was performed with GREAT version 1.8.2 [18]. BEDTools [43] was used for general manipulation of the peak lists, including estimates of overlap between the lists. For motif discovery in the motif enrichment benchmark we used Patser v3e [44], reporting all motifs with score at least equal to the negative value of the sample-size adjusted information content (option -li). TF motif matrices were taken from Jaspar [17], using the default vertebrate matrices for REST, SRF and MAX. Additional matrices were taken from Transfac [45], using the matrices most similar to the Jaspar matrices according to STAMP [46], except for SRF, where the Jaspar matrix has relatively low performance (see Additional file 2: Figure S2). Here the Transfac matrix V$SRF_01 is the one most similar to Jaspar SRF, but the alternative Transfac matrix V$SRF_Q6 showed better performance, and was therefore preferred. For *de novo* motif discovery, we used CisFinder [47] with CG/AT adjustment (removing spurious repetitive GC-rich patterns) and a minimum enrichment ratio of three. The motifs were compared with the relevant Jaspar [17] entries using the T-Reg Comparator [48], and final motif logos were generated with WebLogo [49].

The Triform algorithm was implemented in R using the IRanges package from Bioconductor [15] The ccf function from the R statistical analysis package was used to find the lag with maximum cross-correlation between the forward and reverse coverage distributions.

## Abbreviations

bp: base pairs; ChIP-Seq: Chromatin immunoprecipitation combined with high-throughput sequencing; ERV: Endogenous retrovirus; FDR: False discovery rate; GO: Gene ontology; LTR: Long terminal repeat; MER: Medium reiteration sequence; NLP: Negative Log10 (P); TF: Transcription factor.

## Competing interests

The authors declare that they have no competing interests.

## Authors’ contributions

KK developed the Triform method. KK and TH developed the R-implementation. MBR performed peak profile benchmarking. FD did the motif enrichment benchmark, evaluated the Triform results and drafted the manuscript. All authors contributed to and approved the final manuscript.

## Supplementary Material

Additional file 1 **Supplementary Information.** Implementation of the Triform algorithm. Click here for file

Additional file 2 **Full results of motif enrichment benchmark test.Figure S1** - Results for NRSF. **Figure S2** - Results for SRF. **Figure S3** - Results for MAX. Click here for file
