# Supplementary material for: The Triform algorithm: improved sensitivity and specificity in ChIP-Seq peak finding
Source: BMC Bioinformatics. 2012 Jul 24;13:176. doi: 10.1186/1471-2105-13-176 (PMC3480842; doi:10.1186/1471-2105-13-176)
Supplement: Additional file 1 — Supplementary Information. Implementation of the Triform algorithm. [file 1471-2105-13-176-S1.pdf]

Additional file 1

Implementation of the Triform algorithm

This section gives implementation details for the Triform algorithm. Please see the main paper for tables and equations.

The Triform method was implemented in R using the IRanges package. First the mapped reads are preprocessed as follows:

- Mapped reads for each sample are imported into RangedData objects using symmetrically extended start and end values separated by the predetermined width w (Table 1).
- Each RangedData object is used to produce a set of Rle (Run-length encoding) objects containing strand-specific coverage profiles for each chromosome. These Rle objects supply $C(x)$ and $B(x)$ values for subsequent statistical calculations according to equations (4) - (6) and (8) - (9).
- Each C(x) Rle object is used to produce flanking $C(x - \delta)$ and $C(x + \delta)$ Rle objects, shifted by the predetermined offset δ (Table 1).

Then the Triform algorithm analyzes preprocessed Rle objects as follows:

- All statistical calculations described by equations (4) - (6) and (8) - (9) are performed directly on the associated Rle objects, yielding a set of statistical Rle objects. To avoid division by zero, calculated ratios are set to zero wherever the numerators are zero.
- RleViews objects, specifying all candidate peak regions, are derived from the three statistical Rle objects computed according to equations (4) - (6) by applying the slice function with predetermined lower limit `min.z` (Table 1 and Figure 1b).
- The RleViews objects are filtered to satisfy all of the above specified necessary and sufficient conditions for detecting local peak-like forms on one strand.
- The filtered RleViews objects are converted to IRanges objects specifying all candidate peak regions on each strand.
- The findOverlaps function is used to produce double-stranded IRanges objects specifying all overlapping candidate peak regions on both strands.
- For calculation of local inter-strand lags, the ccf function is applied to $C(x)$ Rle objects within extended ranges of double-stranded candidate peak regions (Figure 1c).
- A final set of double-stranded IRanges objects, specifying all detected peak regions, is obtained by filtering double-stranded IRanges objects according to the above specified criteria for detecting local peak-like forms on both strands.
- For each detected peak region, the `PEAK.LOC` is reported as the midpoint of the range and the `PEAK.NLP` is reported as the sum of the NLP (Negative Log10 (P)) values for the test statistics calculated according to equations (4) - (6).
