# Supplementary material for: The Triform algorithm: improved sensitivity and specificity in ChIP-Seq peak finding
Source: BMC Bioinformatics. 2012 Jul 24;13:176. doi: 10.1186/1471-2105-13-176 (PMC3480842; doi:10.1186/1471-2105-13-176)
Supplement: Additional file 2 — Full results of motif enrichment benchmark test.Figure S1 - Results for NRSF. Figure S2 - Results for SRF. Figure S3 - Results for MAX. [file 1471-2105-13-176-S2.pdf]

Additional file 2

Full results of motif enrichment benchmark test

Please see main paper for details, in particular Figure 3 and the Methods section. Figure 3 of the main paper is taken from Figure S3.

Figure S1 Results for NRSF

Figure S2 Results for SRF

Figure S3 Results for MAX

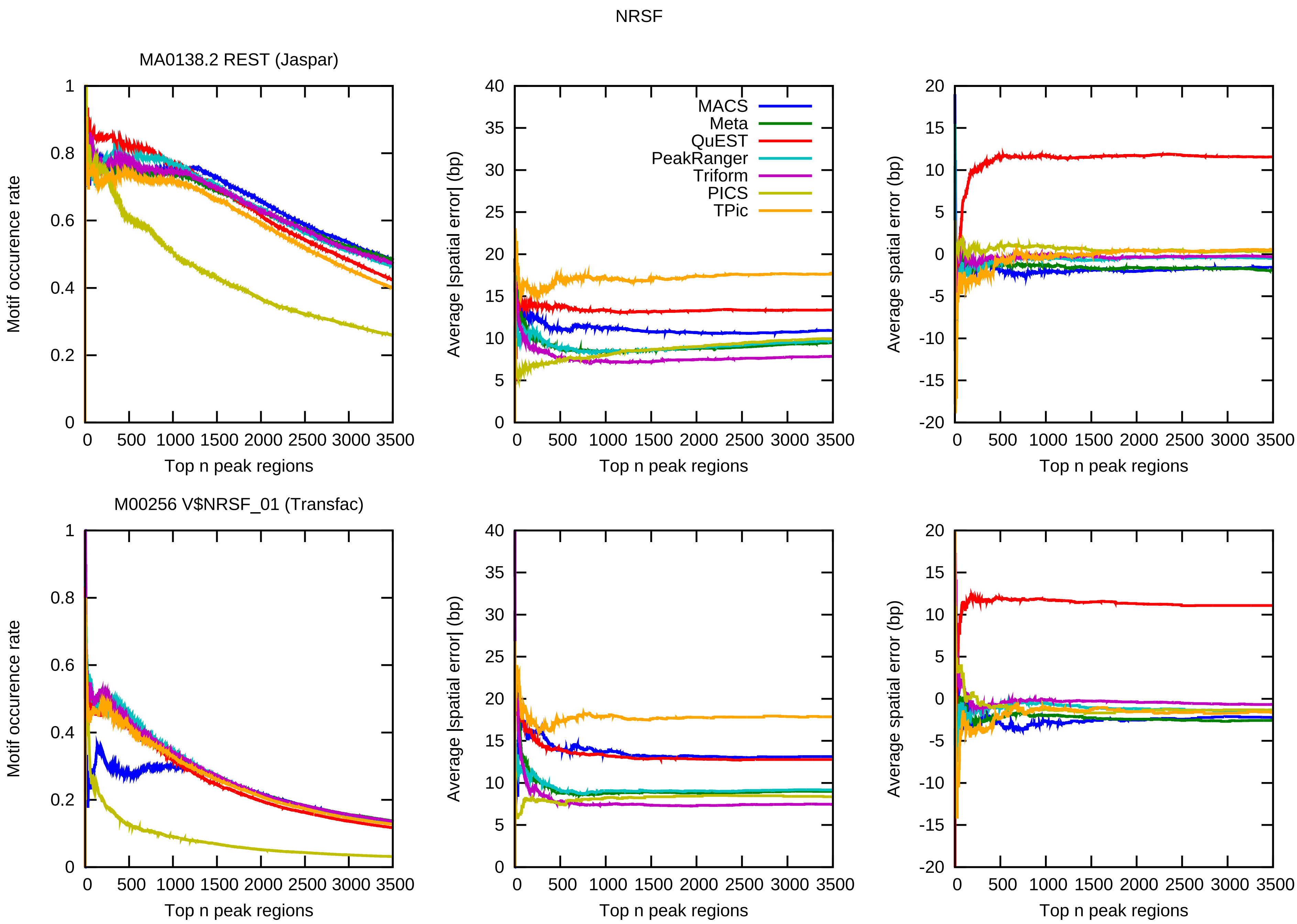

NRSF
MA0138.2 REST (Jaspar)
M00256 V$NRSF_01 (Transfac)
Motif occurence rate
Average |spatial error| (bp)
Average spatial error (bp)
Top n peak regions
MACS
Meta
QuEST
PeakRanger
Triform
PICS
TPic
0
0.2
0.4
0.6
0.8
1
0
5
10
15
20
25
30
35
40
-20
-15
-10
-5
0
5
10
15
20
0
500
1000
1500
2000
2500
3000
3500

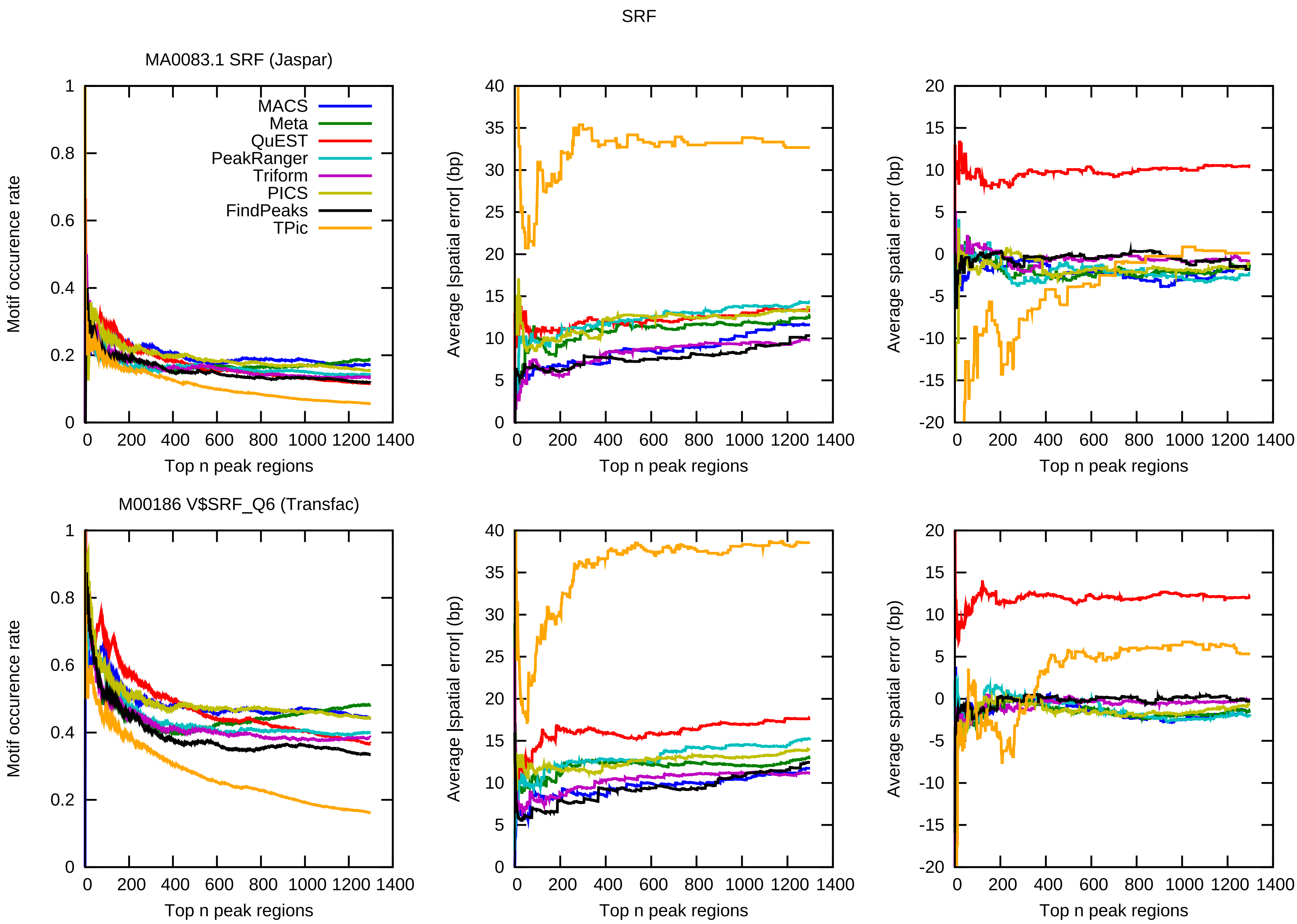
SRF
MA0083.1 SRF (Jaspar)
MACS
Meta
QuEST
PeakRanger
Triform
PICS
FindPeaks
TPic
Motif occurence rate
Average |spatial error| (bp)
Average spatial error (bp)
Top n peak regions
M00186 V$SRF_Q6 (Transfac)

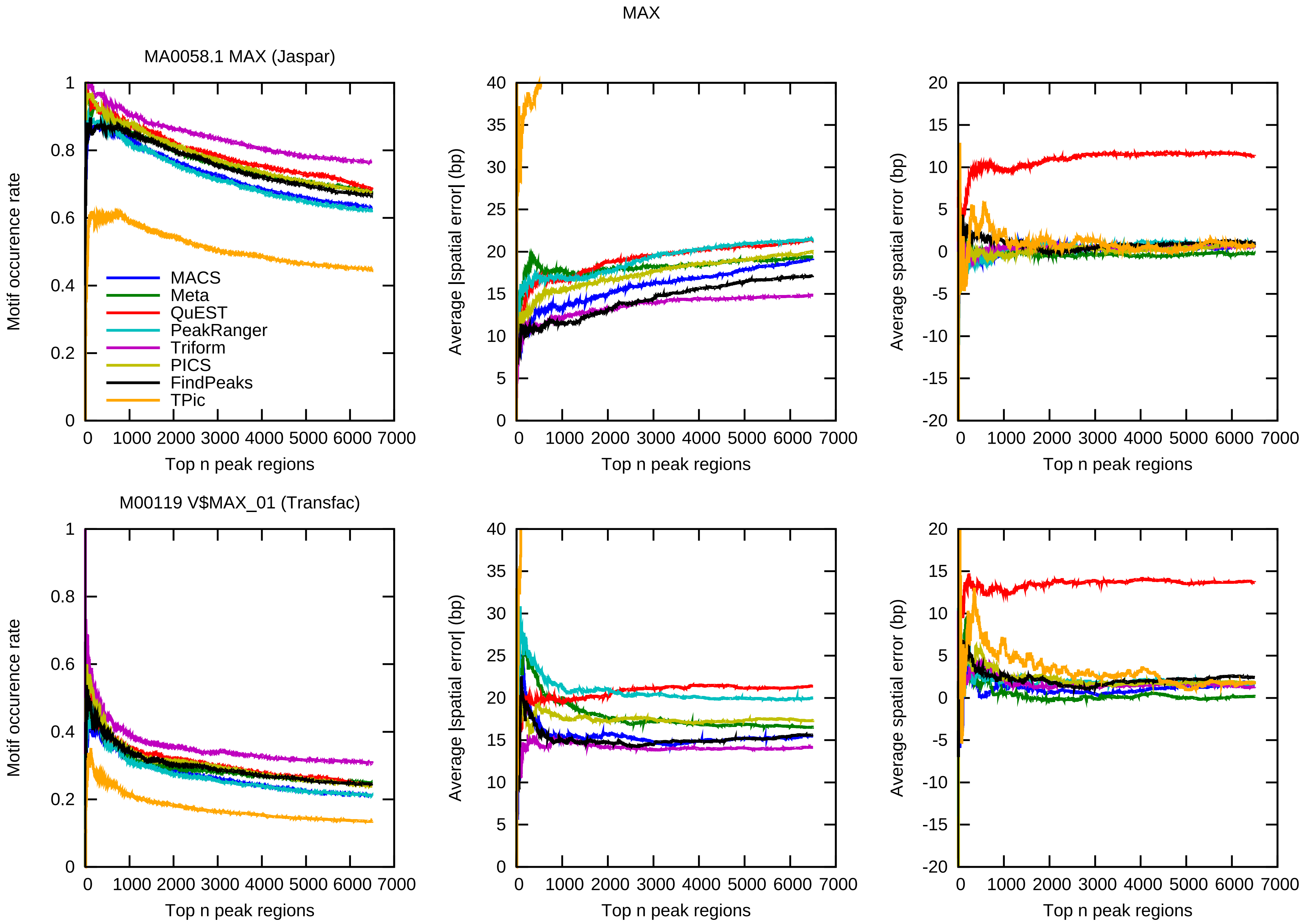
MAX
MA0058.1 MAX (Jaspar)
M00119 V$MAX_01 (Transfac)
Motif occurence rate
Average |spatial error| (bp)
Average spatial error (bp)
Top n peak regions
MACS
Meta
QuEST
PeakRanger
Triform
PICS
FindPeaks
TPic
